# Longitudinal Evaluation Using Preclinical 7T-Magnetic Resonance Imaging/Spectroscopy on Prenatally Dose-Dependent Alcohol-Exposed Rats

**DOI:** 10.3390/metabo13040527

**Published:** 2023-04-06

**Authors:** Tensei Nakano, Tomohiro Natsuyama, Naoki Tsuji, Nanami Katayama, Junpei Ueda, Shigeyoshi Saito

**Affiliations:** 1 Department of Medical Physics and Engineering, Division of Health Sciences, Osaka University Graduate School of Medicine, Suita, Osaka 560-0871, Japan; 2 Course of Medical Physics and Engineering, School of Allied Health Sciences, Osaka University, Osaka 565-0871, Japan; 3 Department of Advanced Medical Technologies, National Cardiovascular and Cerebral Research Center, Suita, Osaka 564-8565, Japan

**Keywords:** preclinical 7T-MRI/MRS, prenatally alcohol-exposed, fetal alcohol spectrum disorder

## Abstract

Prenatal alcohol exposure causes many detrimental alcohol-induced defects in children, collectively known as fetal alcohol spectrum disorders (FASD). This study aimed to evaluate a rat model of FASD, in which alcohol was administered at progressively increasing doses during late pregnancy, using preclinical magnetic resonance (MR) imaging (MRI) and MR spectroscopy (MRS). Wistar rats were orally administered 2.5 mL/day of ethanol (25% concentration) on gestational day 15, and postnatal fetuses were used as FASD models. Four groups were used: a control group (non-treatment group) and three groups of FASD model rats that received one, two, or four doses of ethanol, respectively, during the embryonic period. Body weight was measured every other week until eight weeks of age. MRI and MRS were performed at 4 and 8 weeks of age. The volume of each brain region was measured using acquired T_2_-weighted images. At 4 weeks of age, body weight and cortex volume were significantly lower in the three FASD model groups (2.5 × 1: 304 ± 6 mm^3^, *p* < 0.05; 2.5 × 2: 302 ± 8 mm^3^, *p* < 0.01; 2.5 × 4: 305 ± 6 mm^3^, *p* < 0.05) than they were in the non-treatment group (non-treatment: 313 ± 6 mm^3^). The FASD model group that received four doses of alcohol (2.5 × 4: 0.72 ± 0.09, *p* < 0.05) had lower Taurine/Cr values than the non-treatment group did (non-treatment: 0.91 ± 0.15), an effect that continued at 8 weeks of age (non-treatment: 0.63 ± 0.09; 2.5 × 4: 0.52 ± 0.09, *p* < 0.05). This study is the first to assess brain metabolites and volume over time using MRI and MRS. Decreases in brain volume and taurine levels were observed at 4 and 8 weeks of age, suggesting that the effects of alcohol persisted beyond adulthood.

## 1. Introduction

Fetal alcohol spectrum disorder (FASD) results from prenatal alcohol exposure and causes craniofacial abnormalities, growth retardation, neurological abnormalities, cognitive impairments, and congenital disabilities [1]. The global prevalence of alcohol use during pregnancy is 8–9%. It is also estimated that one in sixty-seven women who consume alcohol during pregnancy will give birth to a child with FASD [2]. However, due to low awareness and diagnostic complexity, FASD remains difficult to diagnose and is often misdiagnosed [3,4]. The effects of prenatal alcohol exposure vary depending on factors such as the amount of alcohol exposure, the duration of administration, and the timing of pregnancy; however, controlling for this uncertainty in animal models is possible. In addition, most FASD animal models are based on early pregnancy (alcohol administration on gestational day 7), whereas a few FASD studies have been conducted where rats were administered alcohol on gestational day 15, corresponding to late gestation [5,6].

Previous studies have reported microcephaly and disproportionate reductions of the basal ganglia volume in patients with FASD using magnetic resonance (MR) imaging (MRI). The effects of fetal alcohol exposure on the brain include generalized growth retardation, as reflected in microcephaly, and characteristic changes in the shape of certain tissues and structural growth of the brain [7]. Alcohol exposure during fetal life has been reported to induce hydrocephalus. In a case, fetal rats treated with 5% (*w*/*v*) ethanol between 10 and 21 days of gestation showed obvious hydrocephalus, characterized by marked enlargement of the lateral ventricles. The T_2_ value (T_2_ relaxation time) obtained from T_2_map images on MRI can detect increased spinal fluid caused by hydrocephalus [8,9].

MR spectroscopy (MRS) is a non-invasive neuroimaging technique that quantifies various neurochemicals [10]. By comparing adolescents diagnosed with FASD and microcephaly with controls, FASD patients had lower N-acetyl aspartate (NAA)/Choline (Cho) and/or NAA/creatinine (Cr) levels in the parietal and frontal cortices, frontal white matter, and corpus callosum than the controls did [11]. However, increased NAA levels have also been reported [12,13]. The FASD study may be because the results depend on various factors, including gestational age at which alcohol was administered, dosage, and the timing and location of brain metabolite measurements. Previous studies using animal models have reported decreased taurine and NAA levels in the cerebellum and striatum of neonates exposed to alcohol [14]. Taurine is an osmotic substance widely present in humans that acts as an antioxidant [15,16,17] and has been suggested to act as a neuroprotector against fetal alcohol exposure [18]. Recent studies have shown that it is a counteractant against mitochondrial dysfunction, endoplasmic reticulum stress, neuroinflammation, synapse loss, and subsequent neuronal cell death, which are major causes of brain disease development [19,20,21]. The dilution and enrichment of taurine concentrations also minimize excessive fluctuations in the cell volume that threaten structural integrity and cellular function [22,23]. This study aimed to evaluate the effects of progressively increasing doses of alcohol administered during late gestation in a rat model of FASD using preclinical high-field 7T-MRI at 4 and 8 weeks of age. MRS was used for the in vivo quantification of brain metabolites.

## 2. Materials and Methods

### 2.1. Animal Preparation

All experimental protocols were approved by the Research Ethics Committee of our University. All experimental procedures involving animals and their care were performed in accordance with Osaka University Guidelines for Animal Experimentation (R02-05-0) and the National Institutes of Health Guide for the Care and Use of Laboratory Animals. Animal experiments were performed on female Wistar rats at 15 days of gestation, purchased from Japan SLC (Hamamatsu, Japan). All rats were housed in a controlled vivarium environment (24 °C; 12:12 h light/dark cycle) and were fed a standard pellet diet and water ad libitum.

### 2.2. FASD Model

Wistar rats were orally administered 2.5 mL/day of ethanol (25% concentration) on gestational day 15, and postnatal fetuses were used as the FASD model. The rats were anesthetized with a mixture of air and 3% isoflurane gas (Wako Pure Chemical Industries, Ltd., Osaka, Japan), and then orally administered ethanol using the sonde method. The female rats were sacrificed 2 weeks after the birth of the fetuses, which were weaned at 3 weeks, with three male rats per gauge. Four groups were used: three FASD model groups that received one (2.5 × 1), two (2.5 × 2), and four doses (2.5 × 4) of alcohol in utero, respectively, and a non-treatment group. The four groups were compared using MRI at 4 and 8 weeks of age (non-treatment: n = 11, 2.5 × 1: n = 11, 2.5 × 2: n = 7, and 2.5 × 4: n = 11).

Weights were postnatally recorded every other week from 2 to 8 weeks, and averages were recorded. Controls were groups in a range of 6~19 animals per group for weight measurements, with female rats included in the measurements being 2 weeks old.

### 2.3. Magnetic Resonance Imaging

MR images of the animal brains were acquired using a horizontal 7T scanner (PharmaScan 70/16 US; Bruker Biospin, Ettlingen, Germany) with a volume coil with an inner diameter of 40 mm. The rats were positioned in a stereotaxic frame to obtain MR images, with their mouths fixed to prevent movement during acquisition [24]. The rats’ body temperature was maintained at 36.5 °C with regulated water flow and was continuously monitored using a physiological monitoring system (SA Instruments Inc., Stony Book, NY, USA). All brain MR experiments on rats were performed under general anesthesia with isoflurane (3.0% for induction and 2.0% for maintenance).

### 2.4. T_2_-Weighted Images Brain Volume

Horizontal cross-sectional T_2_-weighted images (T_2_WI) were acquired using the Turbo Rapid Acquisition with the Relaxation Enhancement (RARE) sequence with the following parameters: repetition time (TR)/echo time (TE) = 3200/32.7 ms; the number of slices = 20; RARE factor = 8; the number of averages = 4; field of view = 36 × 36 mm^2^; matrix size = 256 × 256; slice thickness = 0.5 mm; scan time = 6 min 49 s.

### 2.5. T_2_ Relaxation Time

T_2_ map was acquired using Multi Slice Multi Echo (MSME), and TR/TE = 2250/8.3 ms; the number of slices = 20; echo = 12 echoes; the number of averages = 1; field of view = 36 × 36 mm^2^; matrix size = 256 × 256; slice thickness = 0.5 mm; scan time = 9 min 36 s.

### 2.6. Magnetic Resonance Spectroscopy (MRS) for Brain Metabolites

T_2_WIs were used for MRS to precisely locate 3 × 3 × 3 mm^3^ voxels in the parietal and frontal cortices. Magnetic field uniformity was obtained using a fast, automated shimming technique with mapping along the projection (MAPSHIM) sequence, and good shimming was achieved in voxels (8.9–12.1 Hz). MRS acquisition was performed using a point-resolved spectroscopy (PRESS) sequence (TR/TE = 2500/16.6 ms) combined with variable-power RF pulses with optimized relaxation delay (VAPOR) water suppression. Metabolite spectra were acquired 256 times with VAPOR and 32 times without VAPOR for a total scan time of 10 min and 40 s. Glutamine, glutathione (GSH) myoinositol (mIns), N-acetyl aspartate (NAA), Taurine (Tau), glycerophosphocholine + phosphocholine (GPC + PCh), N-acetyl aspartate + N-acetyl aspartyl glutamate (NAA + NAAG), creatine + phosphocreatine (Cr + PCr), glutamine + glutamic acid (Glu + Gln), and metabolite concentrations were quantified using the LCmodel basis dataset [25].

### 2.7. Image Analysis and Statistical Analysis

The volume of each brain region was defined as the total area of T_2_WI, with four slices showing the tissue multiplied by the slice thickness (0.5 mm). T_2_ values were calculated by placing the region of interest on the T_2_ map. Brain metabolites were standardized as Cr + PCr (the quantitative values of each metabolite were divided by the Cr + PCr value).

Data are presented as mean ± standard deviation (SD). Between-group differences in the estimated values of the parameters, including body weight, the volume of each brain region, T_2_ value, and MRS brain metabolites, were analyzed using Tukey’s multiple comparison test and one-way analysis of variance using Prism 9 (Version 9; GraphPad Software, San Diego, CA, USA). Statistical significance was set at *p* < 0.05.

## 3. Results

### 3.1. Body Weight

**Figure 1:**
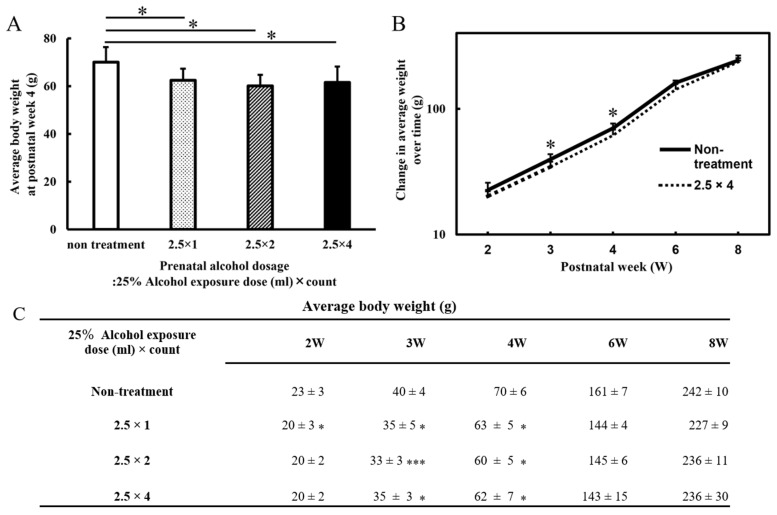
The graph of body weight (g) at postnatal week 4 (postnatal days 28) (**A**). The graph of body weight (g) from the second postnatal week (postnatal days 14) to adulthood (postnatal days 56) (**B**). (**A**) The graph is a four-group comparison (n = 7~11) of the non-treatment, 2.5 × 1, 2.5 × 2, and 2.5 × 4 groups. All FASD groups showed the same weight loss compared with that of the non-treated group at 4 weeks of age (*p* < 0.05). (**B**) Comparison between the non-treated and four-dose alcohol groups (2.5 × 4: n = 11). At 3 and 4 weeks of age, the four-dose group had lower body weights than the control group did; no significant differences were observed at 8 weeks of age (*p* < 0.05). (**C**) The table of body weight (g) from the second postnatal week (p14) to adulthood (p56). Low body weights were observed in all FASD groups compared with controls at 3 and 4 weeks of age. Non-treatment = normal control group; 2.5 × 1 = one dose of 2.5 mL of 25% alcohol; 2.5 × 2 = two doses of 2.5 mL of 25% alcohol; 2.5 × 4 = four doses of 2.5 mL of 25% alcohol. Statistical analysis of a total of four groups (FASD three groups and non-treatment) was performed for each week of age. The results showed that there were no significant differences among the three FASD groups, and there was only a significant difference between the non-treatment and the respective FASD groups. Therefore, the FASD group that was significantly different to the non-treatment group is indicated with an asterisk (compared with the non-treatment: * *p* < 0.05; *** *p* < 0.001).

Body weights were measured from 2 to 8 weeks postnatally, and the averages were recorded (Figure 1). At 3 and 4 weeks of age, the three FASD groups (at 4 weeks: 2.5 × 1: 63 ± 5 g, 2.5 × 2: 60 ± 5 g, and 2.5 × 4: 62 ± 7 g) showed a decrease in body weight compared with that of the non-treatment group (non-treatment: 70 ± 6 g; at 4 weeks: 2.5 × 1: *p* < 0.05, 2.5 × 2: *p* < 0.05, and 2.5 × 4: *p* < 0.05). There was no significant difference in alcohol volume dependence among the three groups. At 2, 6, and 8 weeks of age, all FASD groups did not differ significantly in body weight compared with that of the control group (non-treatment at 8 weeks: 242 ± 10 g, 2.5 × 1: 227 ± 9 g, 2.5 × 2: 236 ± 11 g, and 2.5 × 4: 236 ± 30 g).

### 3.2. Brain Volumes

**Figure 2:**
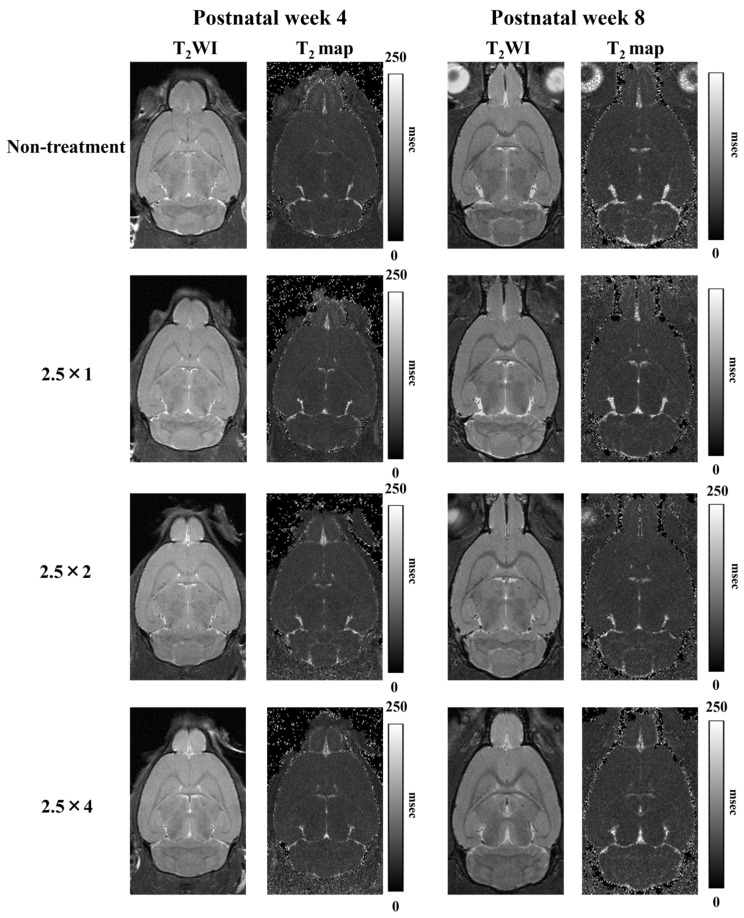
T_2_-weighted and T_2_ map images at 4 and 8 weeks postnatally. The 10th slice of the 20 horizontal cross-sectional images acquired is shown.

The T_2_WI used to measure the volume of each brain region and the T_2_ map images used to measure the T_2_ values are shown in Figure 2. Comparison of the cerebral volumes at 4 weeks showed that the three FASD groups (2.5 × 1: 304 ± 6 mm^3^, 2.5 × 2: 302 ± 8 mm^3^, and 2.5 × 4: 305 ± 6 mm^3^) showed a more significant decrease compared with that of the non-treatment group (non-treatment: 313 ± 6 mm^3^; at 4 weeks: 2.5 × 1: *p* < 0.05, 2.5 × 2: *p* < 0.01, and 2.5 × 4: *p* < 0.05). Similarly, at 8 weeks, the three FASD groups (2.5 × 1: 350 ± 5 mm^3^, 2.5 × 2: 353 ± 6 mm^3^, 2.5 × 4: 352 ± 5 mm^3^) showed a more significant decrease compared with that of the non-treatment group (non-treatment: 361 ± 4 mm^3^; at 8 weeks: 2.5 × 1: *p* < 0.001, 2.5 × 2: *p* < 0.05, and 2.5 × 4: *p* < 0.01). There was no significant difference in alcohol volume dependence among the three groups.

**Figure 3:**
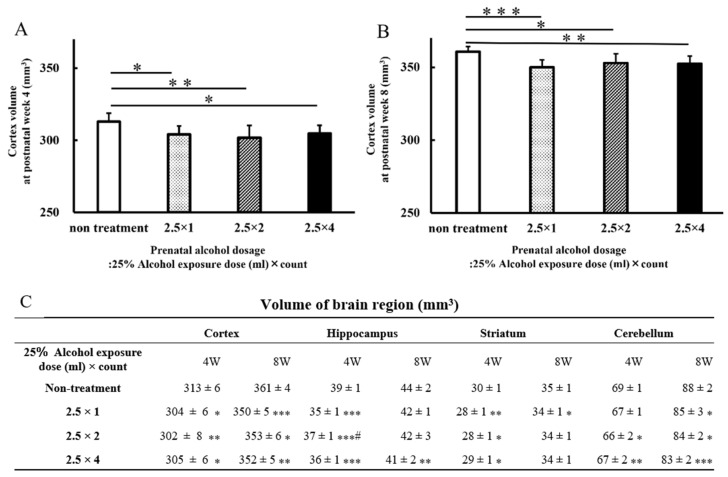
Graphs of postnatal cerebral volume at 4 weeks (**A**) and 8 weeks (**B**). Brain volumes for each brain area at 4 and 8 weeks postnatally: cortex, hippocampus, striatum, and cerebellum (**C**). In the FASD group that received alcohol, there was a larger decrease in brain volume in each brain region compared with that of the non-treated group. Brain volume reduction of the brain regions occurred at 4 and 8 weeks of age. Non-treatment = normal control group (n = 11); 2.5 × 1 = one dose of 2.5 mL of 25% alcohol (n = 11); 2.5 × 2 = two doses of 2.5 mL of 25% alcohol (n = 7); 2.5 × 4 = four doses of 2.5 mL of 25% alcohol (n = 11). Between-group comparisons were analyzed by one-way ANOVA with Tukey’s multiple comparison tests using Prism 9. The results showed no significant difference between the two-dose group (2.5 × 2) and the four-dose group (2.5 × 4). As compared with the non-treatment group: * *p* < 0.05; ** *p* < 0.01; *** *p* < 0.001. As with the one-dose FASD group (2.5 × 1): # *p* < 0.05.

The results of analyses, including data on the hippocampus, striatum, and cerebellum, are presented as graphs and tables (Figure 3). As in the cortex, the hippocampus (2.5 × 1: 35 ± 1 mm^3^, *p* < 0.001; 2.5 × 2: 37 ± 1 mm^3^, *p* < 0.001; 2.5 × 4: 36 ± 1 mm^3^, *p* < 0.001) and striatum (2.5 × 1: 28 ± 1 mm^3^, *p* < 0.01; 2.5 × 2: 28 ± 1 mm^3^, *p* < 0.05; 2.5 × 4: 29 ± 1 mm^3^, *p* < 0.05) of rats in the three FASD groups at 4 weeks of age were significantly reduced compared with those of the rats in the non-treatment group (hippocampus: 39 ± 1 mm^3^, striatum: 30 ± 1 mm^3^). Hippocampal volume at 8 weeks of age in the four-dose FASD group (2.5 × 4: 41 ± 2 mm^3^, *p* < 0.01) was more significantly reduced compared with that of the non-treatment group (non-treatment: 44 ± 2 mm^3^). The striatum volume at 8 weeks of age in the one-dose FASD group (2.5 × 1: 34 ± 1 mm^3^, *p* < 0.05) was more significantly reduced compared with that of the non-treatment group (non-treatment: 35 ± 1 mm^3^).

Cerebellum volume at 4 weeks of age was more significantly reduced in the two- and four-dose FASD groups (2.5 × 2: 66 ± 2 mm^3^, *p* < 0.05; 2.5 × 4: 67 ± 2 mm^3^, *p* < 0.01) compared with that of the non-treatment group (non-treatment: 69 ± 1 mm^3^). Cerebellum volume at 8 weeks of age was more significantly reduced in the one-, two-, and four-dose FASD groups (2.5 × 1: 85 ± 3 mm^3^, *p* < 0.05; 2.5 × 2: 84 ± 2 mm^3^, *p* < 0.05; 2.5 × 4: 83 ± 2 mm^3^, *p* < 0.001) compared with that of the non-treatment group (non-treatment: 88 ± 2 mm^3^).

### 3.3. T_2_ Relaxation Time

**Figure 4:**
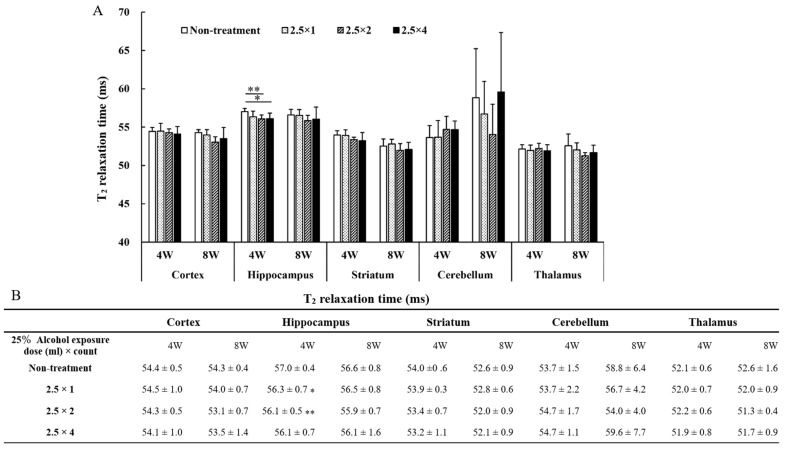
The T_2_ values measured in each brain region are shown in graph (**A**) and table (**B**). Between-group comparisons were analyzed by one-way ANOVA with Tukey’s multiple comparison tests using Prism 9. T_2_ values were lower in the two- and four-dose groups compared with those of the non-treated group (* *p* < 0.05, ** *p* < 0.01). There were no significant differences among the three FASD groups, there was a significant difference only between the non-treatment and the FASD groups (2.5 × 2, 2.5 × 4). Therefore, the FASD group that was significantly different for non-treatment is indicated with an asterisk.

The results of T_2_ values measured in each brain region are shown in the graph and table in Figure 4. The two- and four-dose FASD groups (2.5 × 2: 56.1 ± 0.5 ms, *p* < 0.05; 2.5 × 4: 56.1 ± 0.7 ms, *p* < 0.01) showed a larger decrease in T_2_ values compared with that of the non-treatment group (non-treatment: 57.0 ± 0.4 ms). No significant differences were observed between the cortex, striatum, cerebellum, and thalamus.

### 3.4. MRS Brain Metabolites

**Figure 5:**
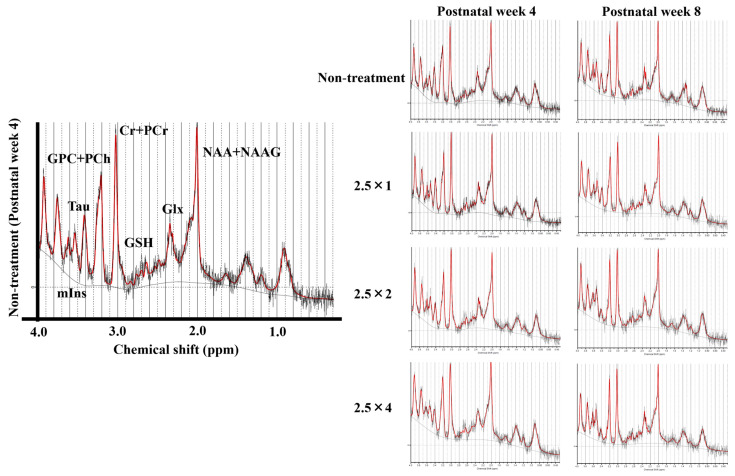
MR spectrum of 4- and 8-week-old rats analyzed using LCmodel.

**Figure 6:**
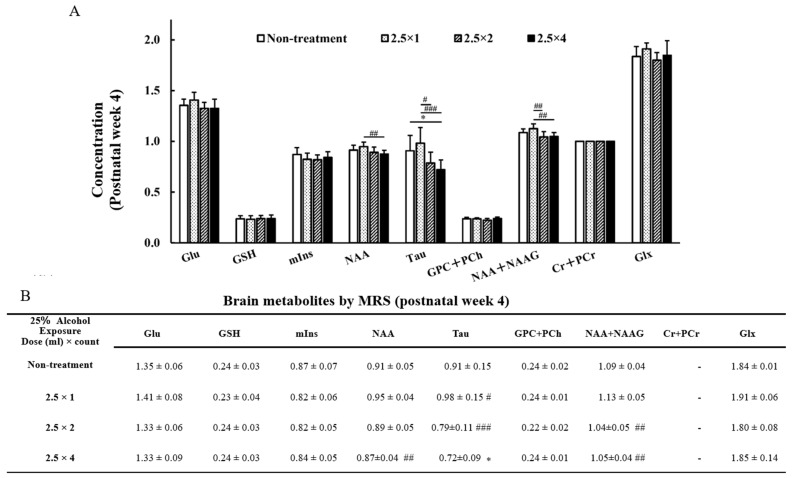
Graph (**A**) and Table (**B**) of brain metabolites on MRS at 4 weeks of age: glutamine (Glu), glutathione (GSH), Myo-inositol (mIns), N-acetyl aspartate (NAA), Taurine (Tau), glycerophosphocholine + phosphocholine (GPC + PCh), N-acetyl aspartate + N-acetyl aspartyl glutamate (NAA + NAAG), creatine + phosphocreatine (Cr + PCr), and glutamine + glutamic acid (Glu + Gln). The results of the comparison between the four-groups (non-treatment, 2.5 × 1, 2.5 × 2, and 2.5 × 4) are shown. Between-group comparisons were analyzed by one-way ANOVA with Tukey’s multiple comparison tests using Prism 9. The results showed no significant difference between the two-dose group (2.5 × 2) and the four-dose group (2.5 × 4). As compared with the non-treatment group: * *p* < 0.05. As compared with the one-dose FASD group (2.5 × 1): # *p* < 0.05; ## *p* < 0.01; ### *p* < 0.001.

**Figure 7:**
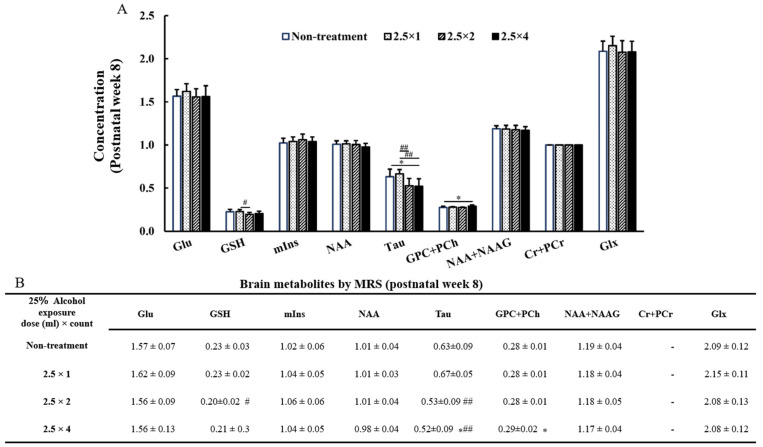
Graph (**A**) and Table (**B**) of brain metabolites on MRS at 8 weeks of age: glutamine (Glu), glutathione (GSH), Myo-inositol (mIns), N-acetyl aspartate (NAA), taurine (Tau), glycerophosphocholine + phosphocholine (GPC + PCh), N-acetyl aspartate + N-acetyl aspartyl glutamate (NAA + NAAG), creatine + phosphocreatine (Cr + PCr), and glutamine + glutamic acid (Glu + Gln). The results of the comparison between the four-groups (non-treatment, 2.5 × 1, 2.5 × 2, and 2.5 × 4) are shown. Between-group comparisons were analyzed by one-way ANOVA with Tukey’s multiple comparison tests using Prism 9. The results showed no significant difference between the two-dose group (2.5 × 2) and the four-dose group (2.5 × 4). When compared with the non-treatment: * *p* < 0.05. When compared with the one-dose FASD group (2.5 × 1): # *p* < 0.05; ## *p* < 0.01.

MR spectra of the thalamus obtained at 4 and 8 weeks of age are shown in Figure 5. The reliable metabolites within 15% SD are shown in the graphs in Figure 6 and Figure 7. By comparing brain metabolites at 4 weeks of age, the four-dose FASD group (2.5 × 4: 0.72 ± 0.09) showed a more significant decrease in Taurine levels compared with those of the non-treatment group (non-treatment: 0.91 ± 0.15, *p* < 0.05) and the one-dose FASD group (2.5 × 1: 0.98 ± 0.15, *p* < 0.001). The two-dose FASD group (2.5 × 2: 0.79 ± 0.11) showed a more significant decrease in Taurine levels than the one-dose FASD group did (2.5 × 1: 0.98 ± 0.15, *p* < 0.05).

By comparing brain metabolites at 8 weeks of age, the four-dose FASD group (2.5 × 4: 0.52 ± 0.09) showed a more significant decrease in Taurine levels compared with those of the non-treatment group (non-treatment:0.63 ± 0.09, *p* < 0.05) and the one-dose FASD group (2.5 × 1: 0.67 ± 0.05, *p* < 0.01). The two-dose FASD group (2.5 × 2:0.53 ± 0.09) showed a larger decrease in Taurine levels than the one-dose FASD group did (2.5 × 1: 0.67 ± 0.05, *p* < 0.01).

**Figure 8:**
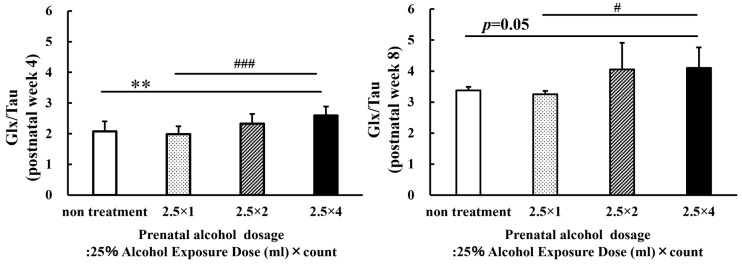
Graphs of the sum (Glx) of glutamine (Glu) and glutamic acid (Gln) were compared with taurine (Glx/Tau). The increase in the Glx/Tau level increased with the alcohol dosage. Between-group comparisons were analyzed by one-way ANOVA with Tukey’s multiple comparison tests using Prism 9. The four-dose group (2.5 × 4) at 4 weeks of age showed a more significant increase in the Glx/Tau level compared with those of the non-treatment group (*p* < 0.01) and the one-dose FASD group (2.5 × 1; *p* < 0.001). The four-dose group (2.5 × 4) at 8 weeks of age showed a more significant increase in the Glx/Tau level than the one-dose FASD group did (2.5 × 1; *p* < 0.05). The results showed no significant difference between the two-dose group (2.5 × 2) and the four-dose group (2.5 × 4). As compared with the non-treatment: ** *p* < 0.01. As compared with the one-dose FASD group (2.5 × 1): # *p* < 0.05; ### *p* < 0.001.

Then, the sum (Glx) of glutamine (Glu) and glutamic acid (Gln) were compared with taurine (Glx/Tau; Figure 8). By comparing Glx/Tau at 4 weeks of age, the four-dose FASD group (2.5 × 4: 2.59 ± 0.30) showed a larger increase compared with those of the non-treatment group (non-treatment: 2.08 ± 0.32, *p* < 0.01) and the one-dose FASD group (2.5 × 1:1.99 ± 0.26, *p* < 0.001). However, by comparing Glx/Tau at 8 weeks of age, the four-dose FASD and control groups (2.5 × 4: 4.10 ± 0.66; non-treatment: 3.38 ± 0.12, *p* = 0.05) showed no significant increase; however, the one-dose FASD group showed an increase (2.5 × 1: 3.25 ± 0.11, *p* < 0.05). Comparisons (statistical analysis) were made between the four groups (non-treatment, 2.5 × 1, 2.5 × 2, and 2.5 × 4) for the growth values of each brain metabolite from 4 W to 8 W, but no significant differences were found due to the considerable variation in both metabolites. Therefore, the data were not included.

### 3.5. NAA + NAAG, NAA at 4 Weeks of Age

By comparing the NAA + NAAG (NAA) changes in the two- and four-dose FASD groups with those in the one-dose FASD group at 4 weeks of age, the two- and four-dose FASD groups (2.5 × 2: 1.04 ± 0.05, *p* < 0.01; 2.5 × 4: 1.05 ± 0.04, *p* < 0.001) showed a larger decrease in NAA + NAAG than the one-dose FASD group did (2.5 × 1: 1.13 ± 0.05). The four-dose FASD group (2.5 × 4:0.87 ± 0.04) showed a larger decrease in NAA than the one-dose FASD group did (2.5 × 1: 0.95 ± 0.04, *p* < 0.01).

### 3.6. GPC + PCh, GSH at 8 Weeks of Age

Significant differences were observed between the GPC + PCh and GSH groups at 8 weeks of age. The four-dose FASD group (2.5 × 4: 0.29 ± 0.02) showed a larger increase in the GPC + PCh level compared with that of the control group (non-treatment: 0.28 ± 0.01, *p* < 0.05). In contrast, the two-dose FASD group (2.5 × 2: 0.20 ± 0.02) showed a larger decrease in the GSH level than the one-dose FASD group did (2.5 × 1: 0.23 ± 0.02, *p* < 0.05).

## 4. Discussion

In this study, a brain developmental assessment of fetal alcohol exposure in late pregnancy was conducted using 7T-MRI. The results showed low body weights and decreased brain volumes at 4 weeks of age. In addition, a decrease in taurine levels, as measured using MRS, was observed at 4 and 8 weeks of age. The decrease in brain volume and taurine levels suggests that these effects persist after adulthood.

### 4.1. Alteration of Body Weight and MRI Parameters in FASD Rats

At 3 and 4 weeks of age, the three FASD groups administered different alcohol doses showed a larger decrease in body weight compared with that of the non-treatment group. However, the low body weight of the FASD groups disappeared as the rats grew older (at 8 weeks after birth). A previous study reported an association between prenatal alcohol consumption and weight loss in rats, with lower body weights occurring when alcohol was administered between gestational days 11 and 22 compared with those of the controls [26]. Alcohol-induced weight differences have been reported to decrease with growth [27]. The possibility that alcohol-exposed mothers’ parenting and feeding behaviors may have influenced their offspring’s low body weight cannot be ruled out [27]. 

In the present study, significant differences in volume were observed in all the brain regions, including the cortex, hippocampus, striatum, and cerebellum. Previous studies reported reduced volumes in the striatum, hippocampus, and cerebellum, which are consistent with the present study’s results [28]. These results suggest the involvement of specific brain regions. This suggests that alcohol consumption affects the entire brain. Shrinkage of the entire brain is speculated to be due to reduced white and gray matter [7]. 

Furthermore, because alcohol-induced hydrocephalus affects the brain volume, we examined whether the FASD model created in the present study demonstrated hydrocephalus. In the present study, the decrease in T_2_ values was localized only in the hippocampus, and no symptoms of hydrocephalus were observed because there was no increase in T_2_ values associated with ventricular enlargement due to alcohol exposure. Previous studies on FASD have reported cases in which hydrocephalus did not occur; however, the details of the disease’s cause remain under investigation [29]. Lower T_2_ values in the hippocampus suggest that alcohol has a stronger effect on the hippocampus than it does on other tissues, as previously described [30].

### 4.2. Brain Metabolites in FASD Rats Measured Using MRS

Alcohol-induced changes in brain metabolism may cause changes in body weight and brain volume, or changes in brain volume may alter the brain metabolite levels [10]. Because MRS can measure brain status in vivo and in real time [31], the effects of alcohol have been frequently studied in humans and animals using MRS [32]. Because the thalamus is a developmentally important tissue [33,34], the effects of alcohol were assessed over time by measuring the content of metabolites in the thalamus. 

The FASD group that received more alcohol (2.5 × 4) showed a larger decrease in taurine levels compared with that of the non-treatment group. This result of decreased taurine is consistent with the administration of alcohol to rats shortly after birth [14]. The FASD group that consumed the smallest amount of alcohol (2.5 × 1) showed no significant difference in the taurine levels compared with that of the non-alcohol group. However, when the amount of alcohol was increased (2.5 × 4), a significant difference was observed. From these data, it can be inferred that the effect is stronger when the amount of alcohol administered exceeds the organism’s tolerance. 

Previous studies have shown that fetal alcohol exposure affects glutamate levels [35]. An MRS study on FASD also reported an effect on glutamate [36,37]. Elevated Gln levels result in osmotic load and oxidative stress, leading to astrocyte dysfunction, following the overproduction of free radicals and the induction of mitochondrial permeability [38]. In contrast, taurine may actively prevent neuronal cell death due to excitatory neurotoxicity by regulating elevated glutamate levels within an acceptable range [39]. In addition, taurine has been reported to inhibit the abnormal motor activity stimulated by alcohol ingestion [40]. Xu, S. et al. reported that certain brain nuclei, following long-term dietary alcohol intake, have elevated Gln and depleted Tau levels indicative of neurochemical changes and behavioral disturbances and considered that metabolites of alcohol produced in the brain and derived from damaged liver may deplete thalamic tau by inducing osmotic and oxidative stress [41]. Additionally, Lunde et al. hypothesized that taurine might normalize glutamate-induced excitatory neurotoxicity in developing brain regions in response to alcohol during pregnancy [18]. The evidence supporting this assumption is that children whose mothers were exposed to alcohol reportedly recovered from the damage to learning and memory regions when their mothers were supplemented with taurine [27]. Therefore, we speculated that the taurine levels would decrease when fetal alcohol exposure exceeded the acceptable limits. In the present study, as hypothesized, the Glx/Tau values increased based on the amount of alcohol consumed. These data indicate the importance of the neuroprotective function of taurine on behavior due to the effects of alcohol. 

NAA is a free amino acid derivative that is second only to glutamate in terms of abundance in the central nervous system. In MRS studies, NAA is a neuronal marker that indicates neuronal survival in various brain injuries [42]. NAA is extracellularly secreted by the cleavage of NAAG, which is extracellularly secreted [42]. There was no significant difference between the non-treatment and FASD groups in the present study; however, the changes in NAA and NAAG levels are consistent with previous studies [14], suggesting that alcohol may affect the metabolism of NAA and NAAG. NAA levels increase rapidly during early brain development [43]. Therefore, the low NAA levels observed in FASD cases may reflect impaired formation during early development.

GPC + PCh is a choline that exists in various forms. Choline, the precursor of acetylcholine, plays a role in memory retention [44]. Decreased choline levels in FASD have been reported in the corpus callosum [45] and cerebellum [37], but not in the thalamus. GSH is a tripeptide composed of three amino acids (glutamic acid, cysteine, and glycine). It plays an auxiliary role in protecting cells from reactive oxygen species such as free radicals and peroxides [46]. The present study’s results showed significant differences in the changes in GPC + PCh levels; however, the increase was small. Because GSH is an antioxidant such as taurine, the decrease in GSH level was within the expected range. However, the change in GPC + PCh and GSH levels was small; therefore, if the number of animals in the non-treated and FASD groups increased, no significant difference would exist. In addition, the lack of a significant difference in GSH level between the non-treated and FASD groups, which were treated four times (2.5 × 4), suggests that the possibility of this change should be carefully examined.

Clinical applications and the future prospect of MRS in FASD will be discussed. Staff in hospitals are not in the habit of performing MRS measurements focusing on taurine for FASD. We believe this is because the amount of taurine that can be measured in humans is lower than it is in rodents and because there have been few large-scale studies of human FASD using MRS. Therefore, it is important to conduct preclinical studies using various FASD models with different amounts, durations, and timing of alcohol administration to accumulate data for clinical application. Our study suggests that among the various alcohol-induced brain metabolites, attention should be focused on taurine. No FASD study using MRS has focused on the relationship between glutamate and taurine as much as this study has. We believe that the results derived from this study are a valuable, albeit partial, part of the overall picture of FASD and can help to provide data to promote MRS for FASD.

### 4.3. Limitations

This study had several limitations. First, the number of included mothers was small. Each FASD group measured in this study consisted of rats (7–11 rats) born to two mothers. This suggests that genetic differences were small and that the variation in measured parameters such as body weight and MRI volume was small. In addition, the MRS in this study measured brain metabolites in a good homogeneous magnetic field (shimming: 8.9–12.1 Hz) by utilizing the MAPSHIM sequence. Since this was a longitudinal study, in which MRS imaging was performed at 4 and 8 weeks of age in rats, we compared the changes from 4 to 8 weeks of age for each metabolite among the four groups. However, there was a large variation, and no significant difference was observed. Therefore, although there are ethical restrictions, it is necessary to increase the number of data by increasing the number of animals. It is important to visualize low-frequency phenomena, evaluate the changes, and make predictions based on probability. Second, we did not measure blood alcohol concentrations during alcohol administration. Because the effects on the fetus were measured only by the amount of alcohol administered, it is necessary to examine the direct effects of alcohol on the fetus from various perspectives. Therefore, it is necessary to analyze the behavior of the FASD group and examine the relationship between brain metabolites and behavior. Third, brain volumes were measured by subjectively observing T_2_WI, and slices in which tissue boundaries could be firmly observed were used as regions of interest. Therefore, the entire tissue volume was not measured in this study. 

## 5. Conclusions

This study examined the developmental effects of fetal alcohol exposure in late pregnancy using 7T-MRI. Significant differences in brain volume and taurine reductions were observed at 4 and 8 weeks of age, suggesting that the effects of alcohol persist into adulthood. This study is the first to assess both brain metabolites and volume over time using MRS. Therefore, preclinical 7T-MRI can be used to evaluate controlled animal models over time and is necessary for future FASD research.

## Data Availability

The data presented in this study are available on request from the corresponding author.
